# National Baby Week

**Published:** 1922-08

**Authors:** 


### NATIONAL BABY WEEK.

THE National Association for the Prevention of Infant Mortality and the National Baby Week Council held on July 3-5 a series of conferences on infant welfare at Carnegie House, 117 Piccadilly. A number of tours to centres, clinics, hospitals and mothercraft classes were arranged.

The first subject taken was the care, including medical attendance, of the child from one to five years old. Dr. Stocks (M.O.H., West Bromwich) assailed the nursery school as injurious to the child because harmful to the mother, whose sense of responsibility and opportunities of learning by experience were taken away. These, on the other hand, were promoted by attendance at mother school and welfare centre. Further, children were crowded together at an age when infection was most common and most dangerous. One well-taught mother was a better asset to a neighbourhood than a whole nursery school. The centre was for the well child ; if it ailed the mother should be advised to call in a doctor or to obtain hospital letters, and to follow the treatment prescribed, which she would more probably do if she had to seek it with effort. He felt it a misfortune that at present few doctors understood the normal healthy child ; that being the case, its management needed guiding by the trained specialist.

Dr. Margaret Hogarth (L.C.C.), who held that the effects of some years of mother-educating now began to show results, did not believe that the nursery school would injure the home. Character training was the little child's great need, and could better be gained from school than from a mother whose temper was upset by fatigue and on whom the child was a parasite. As to epidemics, an object-lesson was given by one recently combated at the Margaret Macmillan School at Deptford, which caused no death and little serious after-effect.

Dr. Brackenbury (British Medical Association) said that infinitely more important than either school or centre was the home. Let all possible causes of the mother's absence be removed ; let us see to it that she had good home, good implements, good training.

Let the centre be a place not of treatment for the child, but of education for the mother, of inspection and good counsel.

Dr. Harold Watkins upheld the value of voluntary societies and workers in visiting homes and bringing mothers to the centres.

Miss Eckhardt, tutor in the Ratan Tata department of Social Science, London University, maintained that mothercraft lessons should not be given to schoolgirls, but deferred till they were about to become mothers themselves; only simple health teaching and sewing for babies should be permitted. Miss Norah March suggested that if lessons were not to be given in school, voluntary associations, such as Girl Guides, might undertake them. Dr. Alice Hutchins added that undoubtedly mistakes were made in mothercraft lessons. A girl of 13 should be taught to keep feeding bottles in order, but it was needless to instruct her in the composition of feeds and expect her to remember this 10 years later; the doctor would then give any needed details.

Dr. Buchan (M.O.H., Willesden) thought that where preventive medicine ended and treatment became curative it was impossible to say. There were 1,960 centres in the country to which children in early stages of enteric or adenoid trouble might be brought and be saved by promptitude from growing worse. All subsequent speakers agreed that immediate help should be given. But all such treatment should be linked with domiciliary care. Mr. Turner, representing the British Medical Association, advised the staffing of centres by experienced men and women. Centres should not be allowed to become infant out-patient departments.

Mr. Arthur Collins, formerly treasurer to the Birmingham Corporation, opening a debate upon "The Advisability of Block Grants," urged the need for careful thought in framing a new scheme for making Government grants towards infant welfare. The present system of making these grants in the form of 50 per cent. of the local expenditure had been condemned by the Geddes Committee; but block grants might be just as uneconomical, and it was easier to criticise than to construct a new basis. His own view was that there should be a schedule of admissible cases qualifying for grant, under each head of service, so as to permit expansion of the work, and to give the advantage to the poorer districts which had a large number of legitimate cases for grant. It was important also that considerable latitude should be allowed to each local authority to use its grants as it thought best, especially in the direction of co-operation with voluntary agencies.

Dr. Moore (Huddersfield) thought that the best means of achieving economy would be the abolition of the grant.

Dr. Daley (Blackburn) advocated a system of block health grants for the whole of the public health services, including tuberculosis, venereal disease, and infant welfare, leaving to each local authority the division of the money.

Dr. Ralph Smedley (West Sussex) said that in county areas it was most important to concentrate public health work, and he had no hesitation in saying that this was best undertaken by the district nurse-midwife, who had unique opportunities of becoming familiar with the circumstances of the people in her area. In 1919 his County Council approved of a scheme for the division of the county into 64 nursing districts, staffed with 81 district nurses. At the present time 55 out of the 64 nursing associations had been formed, and the nurses employed by them numbered 66, covering three-quarters of the county. Last year, in spite of the hot, dry summer, the infant mortality in the rural districts was only 37. There had been a progressive and almost uninterrupted decline in the infant mortality rate for the county from 85 per 1,000 births in 1911 to 49 for the past two years. The total expenditure was negligible when spread over the population of a county, representing in West Sussex, for example, just under 6d. a head. This sum represented about 4½d. per head spent on maternity and child welfare work, and the rest on school work and tuberculosis.

The Hon. Mrs. Eustace Hills, Chairman of the National Society of Day Nurseries, urged that Government grants in support of day nurseries were an economical investment. For women who were obliged to work, the day nursery was the only alternative to parting with the child entirely, or placing it with an inexperienced foster-mother or baby-minder. The day nursery of to-day was very different from the crèche of 30 years ago, at which the babies died like flies. Day nurseries had also a threefold educational value: the education of the mother, the training of probationers as nursery nurses, and as a training ground in which mothercraft classes were held for the senior girls attending local Council schools.

Miss Beavan, of Liverpool, mentioned a case in which the cost of the day nursery was 25s. to 28s. per week per child. It would be better to give this money to the mother and let her stay at home and mind the child.

Dr. Scurfield did not approve of any institution for babies where a good home or good foster-mother could be found. "Send the babies," he said, "if they must be separated, to the country. Don't keep them in the towns."

In a discussion on health visitors, Mrs. Eve (late of the Ministry of Health) suggested that the Ministry of Health should fix a standard of qualification and a minimum salary.

The Earl of Onslow, Parliamentary Secretary to the Ministry of Health, presiding on July 5 at a meeting at the Caxton Hall, said it was satisfactory that the Committee on National Expenditure were able to accomplish their task without drawing on the million odd which was now being spent by the State for the benefit of mothers and children. It was anticipated that in 1922-23 existing services could be maintained for a sum considerably less than they cost in 1921-22. This had enabled the Ministry of Health to promise a grant for some urgent extensions, such as the maintenance of the model infant welfare centres which the Carnegie Trustees had so generously offered at a cost of £25,000 each to Birmingham, Liverpool, Rhondda, and Shoreditch.

Dr. Edgar Collis said that most babies at birth were healthy, and only acquired disablement and ill-health through environment after birth. Expenditure on infant welfare was, therefore, not unproductive philanthrophy. Modern industry in the interest of efficiency was calling for a higher grade of workers physically and mentally. In order to obtain this efficiency attention must first be directed to child life.

In a letter to the Press Mrs. Lloyd George asks all who can to send a cheque in favour of the National Baby Week Council to her in order that the Council may pay off a debt of £600 and raise a fund of £10,000, and pursue an active campaign of life-saving at the least possible cost to the nation.

